# The Oath of Hippocrates

**Published:** 1905-09

**Authors:** 


### Domestic Correspondence.

### THE OATH OF HIPPOCRATES.

BOSTON, May 12, 1905.

TO THE EDITOR:

Sir,—Appended please find the Oath of Hippocrates. This, as you know, is the basis for professional ethics to-day. This is the line in which the dental profession is lacking, I think.

HORACE L. HOWE.

"I swear by Apollo the physician, and Æsculapius, and Health, and All-heal, and all the gods and goddesses, that, according to my ability and judgment, I will keep this Oath and this stipulation—to reckon him who taught me this Art equally dear to me as my parents, to share my substance with him, and relieve his necessities if required; to look upon his offspring in the same footing as my own brothers, and to teach them this art, if they shall wish to learn it, without fee or stipulation; and that by precept, lecture, and every other mode of instruction, I will impart a knowledge of the Art to my own sons, and those of my teachers, and to disciples bound by a stipulation and oath according to the law of medicine, but to none others. I will follow that system of regimen which, according to my ability and judgment, I consider for the benefit of my patients, and abstain from whatever is deleterious and mischievous. I will give no deadly medicine to any one if asked, nor suggest any such counsel; and in like manner I will not give to woman a pessary to produce abortion. With purity and with holiness I will pass my life and practise my Art. I will not cut persons laboring under the stone, but will leave this to be done by men who are practitioners of this work. Into whatever houses I enter, I will go into them for the benefit of the sick, and will abstain from every voluntary act of mischief and corruption; and further, from the seduction of females or males, of freemen and slaves. Whatever, in connection with my professional practice or not in connection with it, I see or hear, in the life of men, which ought not to be spoken of abroad, I will not divulge, as reckoning that all such should be kept secret. While I continue to keep this

Oath unviolated, may it be granted to me to enjoy life and the practice of the Art respected by all men, in all times! But should I trespass and violate this Oath, may the reverse be my lot."—*From "The Genuine Works of Hippocrates," translated by Francis Adams, LL.D.*

